# EGLN1 inhibition reverses angiogenesis impairment in hyperglycemia by activating autophagy

**DOI:** 10.1038/s41598-025-19745-6

**Published:** 2025-10-14

**Authors:** Fengli Hu, Zheng Li, Ying Li, Yaxin Zhi, Ting Tang, Pengfei Wang, Ling Xue

**Affiliations:** Cardiology department, Second Hospital of Hebei Medical University, Shijiazhuang, China

**Keywords:** EGLN1/PHD2, Diabetic cardiomyopathy, Angiogenesis, Autophagy, Cardiology, Diseases, Endocrinology, Molecular medicine

## Abstract

Cardiovascular complications have emerged as the predominant cause of mortality in diabetes mellitus (DM), with diabetic cardiomyopathy (DCM) representing a critical clinical challenge due to its distinct pathogenesis independent of coronary artery disease or hypertension. DCM is characterized by left ventricular diastolic dysfunction, hypertrophy, and fibrosis, with impaired myocardial angiogenesis contributing to adverse cardiovascular outcomes. Despite this, targeted therapies for angiogenesis restoration in DCM remain elusive. Through bioinformatic analysis of public databases, we identified egl-9 family hypoxia inducible factor 1 (*EGLN1*) as a differentially expressed gene in both preclinical DCM models and clinical specimens. To validate its role, we established mice model of diabetes and corresponding endothelial cells cultures under hyperglycemic conditions. Results demonstrated significant upregulation of EGLN1 in diabetic mice, correlating with angiogenesis impairment. Intriguingly, EGLN1 inhibition attenuated high glucose-induced endothelial dysfunction by activating autophagy pathways, as evidenced by increased LC3-II/LC3-I ratios and decreased P62 levels. These findings unveil EGLN1 as a novel regulator of myocardial angiogenesis in DCM, proposing autophagy activation as a potential therapeutic strategy to ameliorate diabetic cardiovascular complications. This study provides the first evidence linking EGLN1 dysregulation to angiogenesis defects in DCM, offering insights for targeted intervention development.

**Supplementary Information:**

The online version contains supplementary material available at 10.1038/s41598-025-19745-6.

## Introduction

As the prevalence of diabetes mellitus (DM) continues to increase, diabetic cardiomyopathy (DCM) has become a key issue in the field of diabetes^1–3^. Unlike other cardiovascular diseases, DCM usually does not have coronary artery disease or hypertension, but is often accompanied by left ventricular diastolic dysfunction, ventricular hypertrophy, and myocardial fibrosis^2,4^. Currently, the study found that the occurrence of DCM involves multiple mechanisms, including myocardial inflammation^5,6^, pyroptosis^6^ and ferroptosis^7–10^. In addition to cardiomyocyte dysfunction, damage to endothelial cells is considered to be the origin of early type 2 diabetes microvascular dysfunction^11^. Previous studies have found that microangiogenesis is affected in diabetes or metabolic disorders^12,13^. Although the treatment of diabetes is becoming more mature, more targeted treatment options for DCM, especially microangiogenesis damage, are urgently needed. With the sharing of large biomedical databases, researchers have identified many key regulatory molecules by integrating genomics, epigenetics, transcriptomics and proteomics data. Some studies have found that the key genes of DCM are enriched in the angiogenesis process^14^, but no studies have been conducted on the analysis of key molecules related to diabetic cardiomyopathy and angiogenesis.

Egl-9 family hypoxia inducible factor 1 (EGLN1) is also known as prolyl hydroxylase domain 2 (PHD2). Studies have confirmed that it regulates its degradation by hydroxylation of hypoxia-inducible factor (HIF)^15^. This mechanism is not only involved in physiological hypoxia adaptation, but is also crucial for embryonic development as well as erythrocythemia, pulmonary hypertension and angiogenesis^16,17^. A recent study combined with bioinformatics and in vitro validation found that upregulation of PHD2 in diabetic cardiomyopathy may be a potential intervention target^18^.

Besides, autophagy is a ubiquitous physiological process that can remove harmful misfolded proteins, damaged organelles and cell debris through lysosomes^19^. Recently, a growing number of studies have pointed out that autophagy is crucial in the pathogenesis of DCM^20,21^. Studies have shown that autophagy is related to the occurrence and development of DCM and is a potential pharmacological target for DCM^22^. By identifying these specific molecules, it not only helps to improve the diagnosis and treatment of diabetic cardiomyopathy^23–25^, but also high-risk patients can be identified by predicting the risk of heart failure^26^. Through comprehensive analysis of sequencing data and public databases, we hope to identify genes related to angiogenesis damage in diabetic cardiomyopathy. Based on this, the effect of this molecule on autophagy pathway and angiogenesis was explored.

## Results

### Differential gene screening associated with diabetic microvascular injury

The GSE43950 dataset contains CD34^+^ cell sequencing results from diabetic patients with microvascular complications and matched healthy people. Among them, GSM1074884, GSM1074885, GSM1074886, GSM1074887, and GSM1074878 are healthy control sequencing data, while GSM1074876, GSM1074877, GSM1074880, GSM1074881, and GSM1074883 are diabetic patients with combined microvascular damage (MVD).

**Fig. 1:**
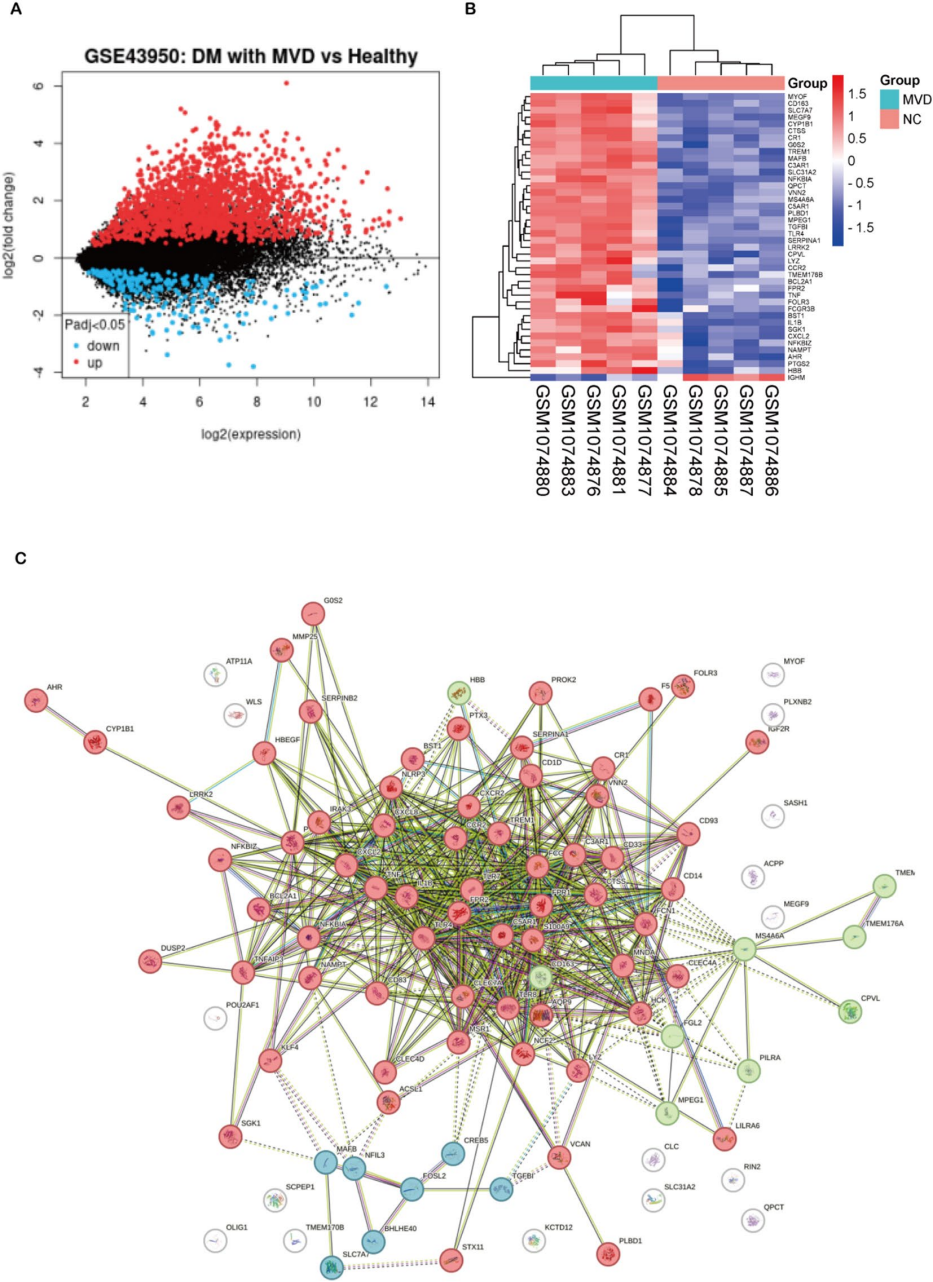
Differential gene screening associated with diabetic microvascular injury. (**A**) Volcano plot of differentially expressed genes from the GSE43950 dataset. (**B**) Heat map of the top 100 differentially expressed genes in GSE43950. (**C**) Protein-protein interaction network diagram of the top 100 differentially expressed genes. K-means clustering was used, where red clustered to immune response-regulating signaling pathway, green clustered to CD20-like family and Cranial nerve maturation. NC, negative control; DM, diabetes mellitus; MVD, Microvascular damage.

Using the online GEO2R for differential analysis, we obtained 1868 differential genes, of which 215 genes were down-regulated and 1653 genes were up-regulated (Fig. 1A). Heat map of the top 100 differentially expressed genes (Fig. 1B), and we found that most of the genes with significant changes in diabetes patients with MVD were upregulated. We then used STRING to perform protein-protein interaction network analysis, and we can see that these genes have a close interaction relationship (Fig. 1C). Moreover, K-means clustering was performed to find that most genes were concentrated in the immune response-regulating signaling pathway.

### Top 100 differentially expressed genes expression and pathway enrichment analysis

**Fig. 2:**
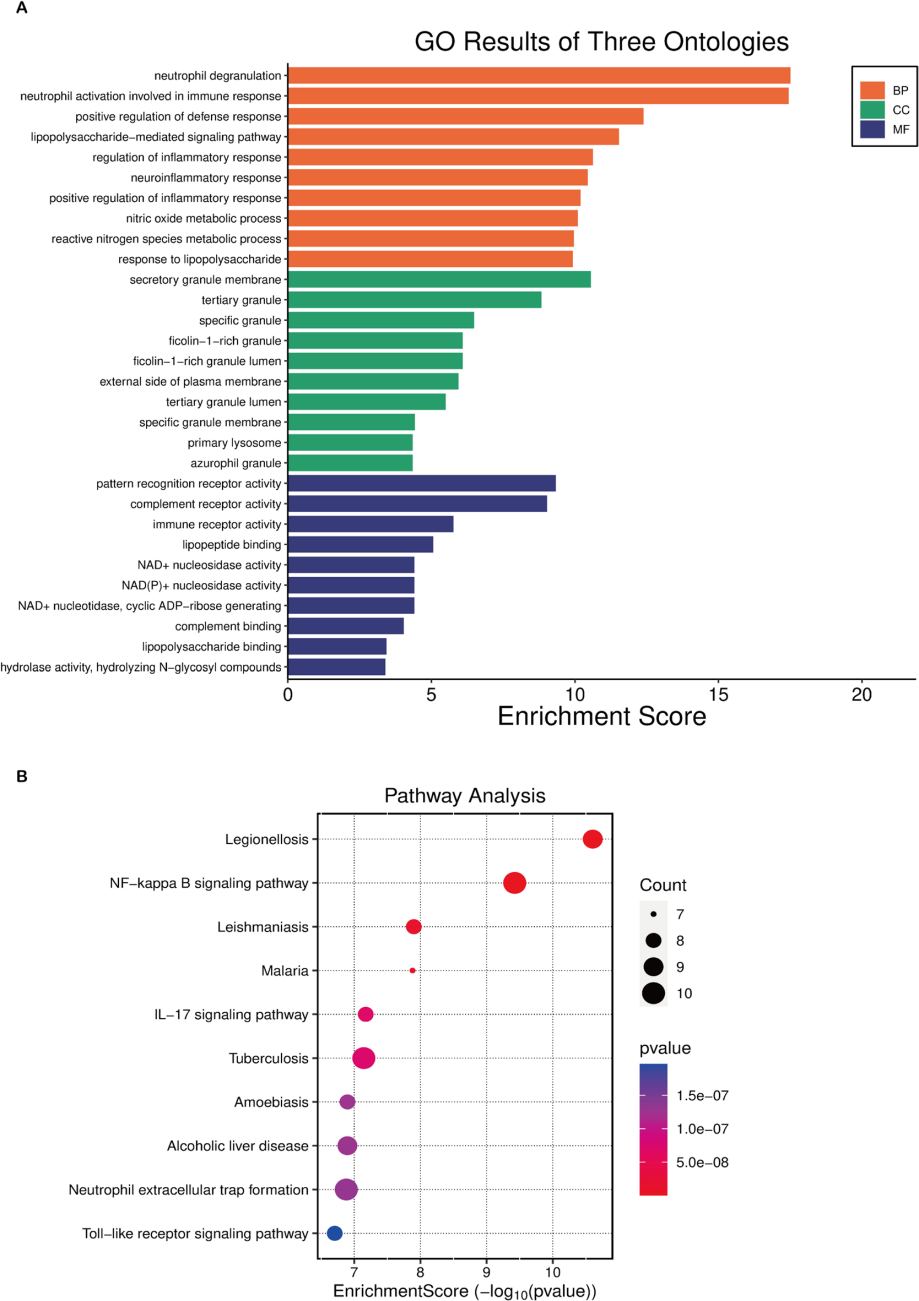
Top 100 differentially expressed genes expression and pathway enrichment analysis. (**A**) GO analysis of the top 100 differentially expressed genes. (**B**) KEGG analysis of the top 100 differentially expressed genes. BP, biological process; CC, cellular component; MF, molecular function. KEGG pathway maps are copyright protected by Kanehisa Laboratories. Used with permission.

In order to explore the role of these differential genes in the physiological and pathological process, we performed GO and KEGG analyses of the top 100 genes with significant differences. In GO analysis (Fig. 2A), these differential genes were enriched in the neutrophil degranulation, neutrophil activation involved in immune response, and positive regulation of defense response. And cellular component included secretory granule membrane, tertiary granule and specific granule. Besides, molecular function mainly enriched in pattern recognition receptor activity, complement receptor activity, and immune receptor activity. KEGG analysis of the top 100 differentially expressed genes showed that these genes were enriched in legionellosis, NF-kappa B signaling pathway and leishmaniasis pathways, and enrichment map suggested that these pathways had mutual influences (Fig. 2B).

### Screening and analysis of key differentially expressed gene

**Fig. 3:**
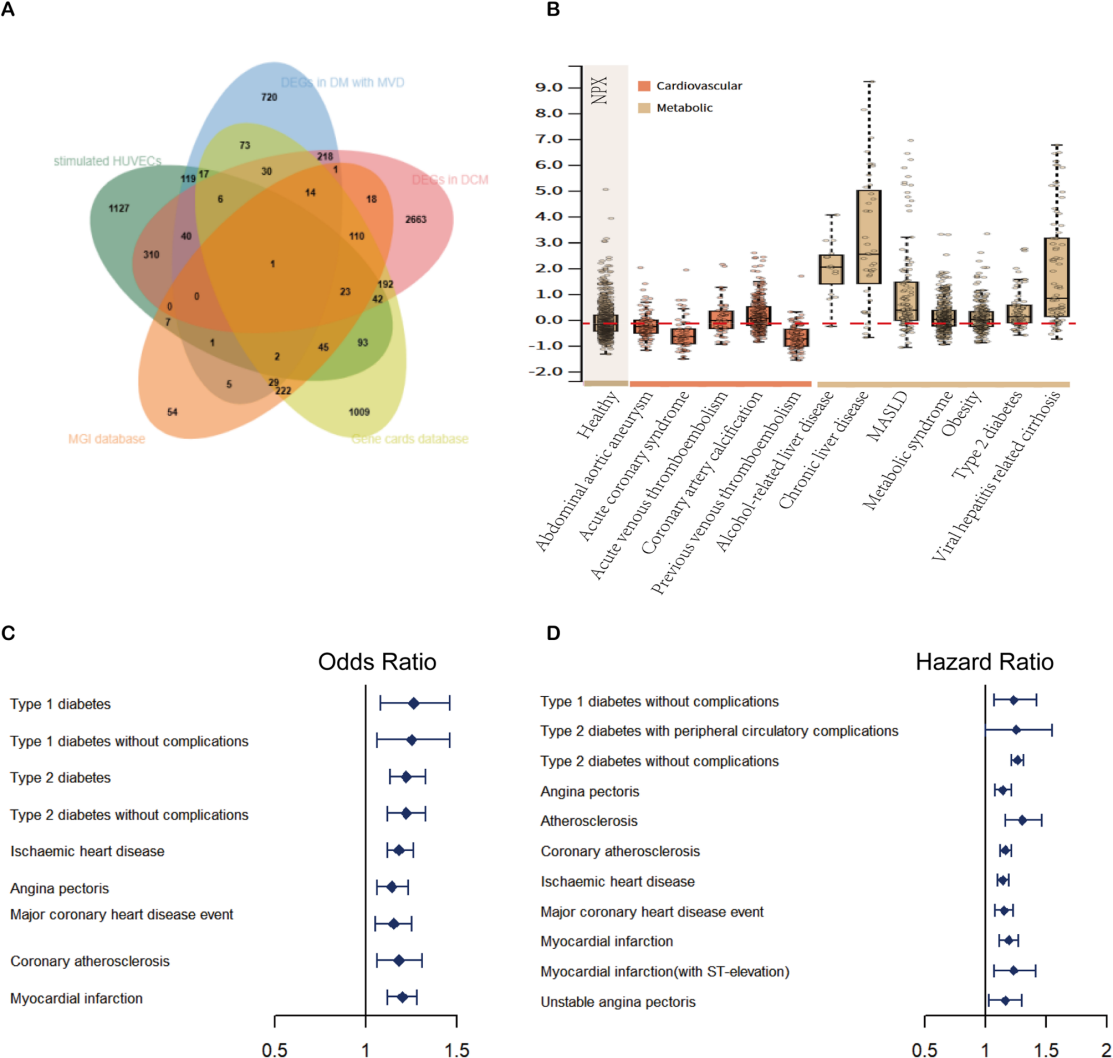
Screening and analysis of key differentially expressed gene. (**A**) Venn diagram of different sequencing datasets and databases to screen differentially expressed genes. (**B**) Box plot of EGLN1 expression levels in metabolic and cardiovascular disease. Data were derived from plasma proteins quantified by proximity extension assay in the HPA database. (**C**) Results of logistic regression analysis of EGLN1 in PPA in metabolic and cardiovascular diseases. (**D**) Results of cox proportional risk regression analysis of EGLN1 in PPA in metabolic and cardiovascular diseases. NPX, normalized protein expression.

Subsequently, three sequencing datasets were intersected with angiogenesis-related genes to identify genes that were different in each database. Finally, we get 1 gene: EGLN1 (Fig. 3A). The expression of EGLN1 in cardiovascular and metabolic diseases was extracted by human protein atlas (HPA), and it was found that the blood tests of patients with type 2 diabetes showed a slight increase in EGLN1, and most metabolic diseases showed an increase (Fig. 3B). Next, we performed a clinical disease risk analysis of EGLN1 in the proteome-phenome atlas(PPA) database, which contains plasma proteomic data from 53,026 people in UK Biobank. Logistic regression (Fig. 3C) and cox regression (Fig. 3D) were used to find that this gene was significantly associated with cardiovascular and metabolic diseases, and this gene may be a risk gene for cardiovascular and metabolic diseases.

### Impaired angiogenesis and upregulation of EGLN in diabetic mouse hearts

**Fig. 4:**
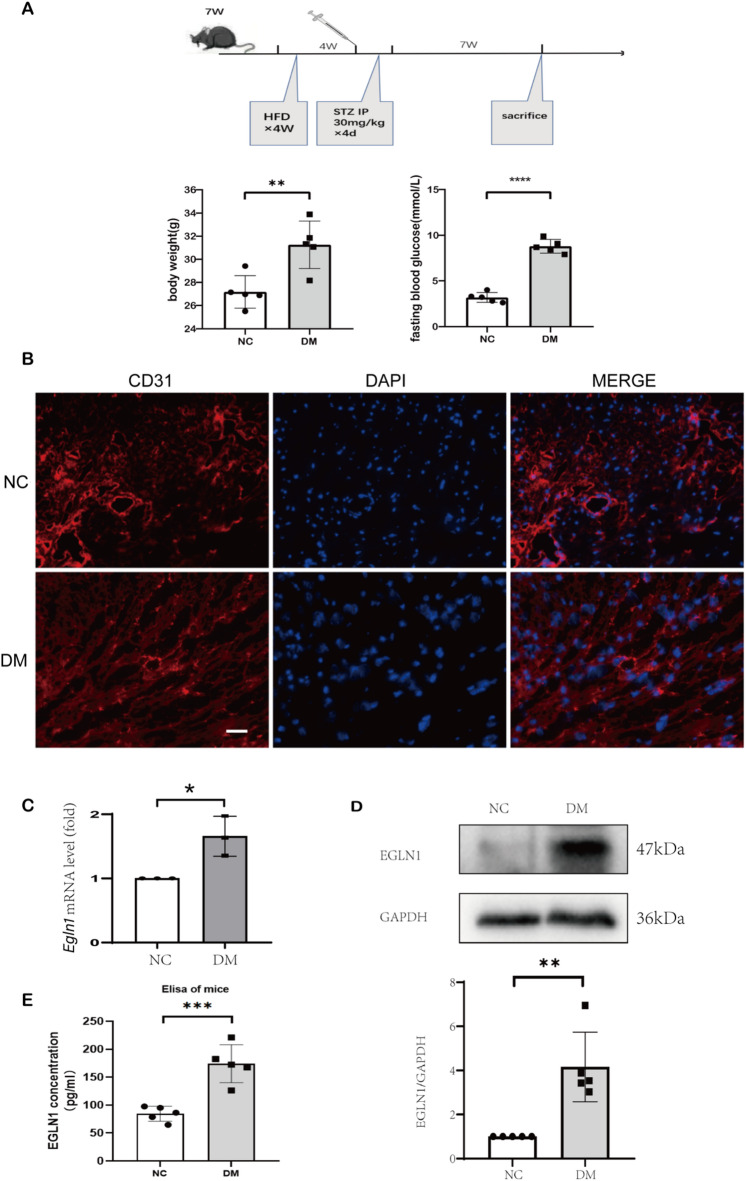
Impaired angiogenesis and upregulation of EGLN in diabetic mouse hearts. (**A**) Pattern diagram of diabetic mouse model and histogram of statistical analysis of body weight and fasting blood glucose. (*n* = 5). (**B**) Confocal image of cardiac microangiogenesis (CD31, red) in mouse heart tissue, scale bar, 50 μm. (**C**) Statistical analysis of qPCR of mice heart tissue. (*n* = 3) (**D**) Representative western blot images and relative analysis of EGLN1 on hearts of NC and DM mice. (*n* = 5). (**E**) ELISA measurements of EGLN1 concentrations in mouse plasma. (*n* = 5). All data represent the mean ± SEM. *P <* 0.05, **P < 0.01, ***P < 0.001, ****P < 0.0001.

To mimic diabetic cardiomyopathy injury, we constructed a mouse model of metabolic disorder/diabetes mellitus (Fig. 4A). After 11 weeks of modeling, the weight and fasting blood glucose of all mice were measured before the mice were sacrificed. Statistical analysis showed that HFD combined with streptozotocin (STZ) had significant effects on body weight and fasting blood glucose in mice (Fig. 4A). As a result, we constructed a mouse model of metabolic disorder/diabetes mellitus similar to the previous study^27^. Subsequently, to assess changes in cardiac microangiogenesis in diabetic mice, we performed immunofluorescence staining of cryosections of cardiac tissue (Fig. 4B). It showed that the CD31 staining of heart microvessels in DM mice was weakened overall, and the continuity of vascular CD31 staining was weakened.

We then performed quantitative real-time polymerase chain reaction (qPCR) and western blot (WB) on mouse hearts to determine how EGLN1 changes in the hearts of diabetic mice (Fig. 4C,D). A significant increase in EGLN1 mRNA and protein levels was observed in the hearts of mice in the DM group, and this increase was statistically significant (*P =* 0.0218, 0.0012 respectively). At the same time, we performed Enzyme-linked immuno sorbent assay (ELISA) assays on the plasma of mice to assess the level of circulating EGLN1 (Fig. 4E). Similar to the western blot assay, we found that EGLN1 in the blood of mice in the DM group also showed a clear increase trend compared to the control group. The mean plasma value of EGLN1 in the NC group was 84.48 pg/mL, while that in the DM group was 174.0 pg/mL. The t-test was used to find that there was a statistically significant difference between groups (*P* = 0.0006).

### EGLN1 upregulation under high glucose suppresses autophagy and impairs angiogenic capacity in human umbilical vein endothelial cells (HUVECs)

**Fig. 5:**
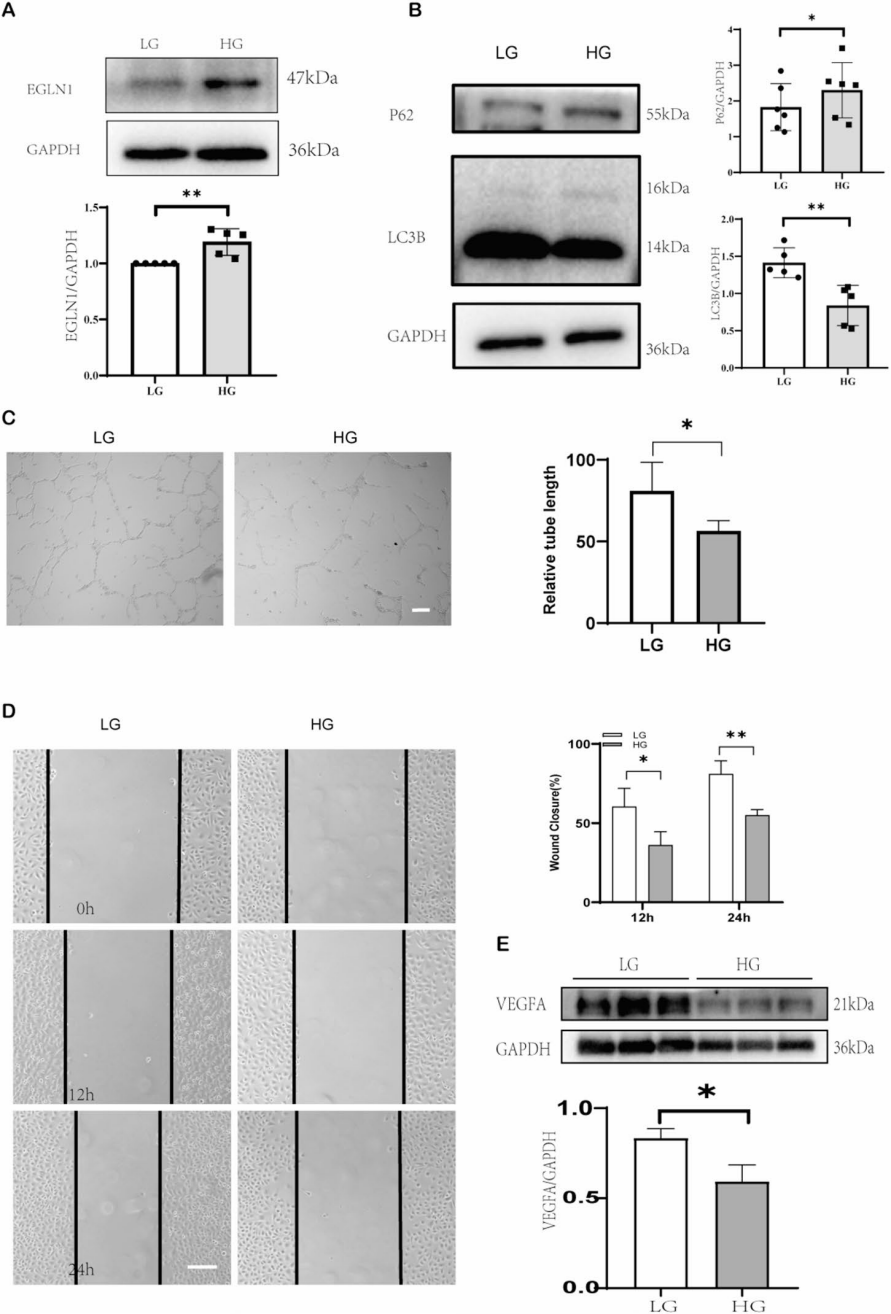
EGLN1 upregulation under high glucose suppresses autophagy and impairs angiogenic capacity in HUVECs. (**A**) Representative western blot images and relative analysis of EGLN1 protein levels in HUVECs exposed to 30 mM glucose for 48 h compared with normal glucose controls (5.5 mM) (*n* = 5). (**B**) Representative western blot images and relative analysis of LC3B-II/LC3B-I ratio (*n* = 5) and P62 (*n* = 6) in high glucose-treated HUVECs (*n* = 3). (**C**) Representative images and quantification of tube formation assay, scale bar = 100 μm (*n* = 3). (**D**) Representative images and quantification of wound healing assay in high glucose-treated HUVECs at 12 and 24 h, scale bar = 100 μm (*n* = 3). (**E**) Western blot images and relative analysis of VEGFA in high glucose-treated HUVECs (*n* = 3). All data represent the mean ± SEM. *P < 0.05, **P < 0.01.

HUVECs were collected after 48 h of normal culture and high glucose stimulation with 30 mmol/L glucose as the high-glucose stimulation condition. A typical western blot of EGLN1 showed that high glucose significantly increased the level of EGLN1 protein in HUVECs (Fig. 5A), and the difference was statistically significant in multiple replicates (*P =* 0.0069). microtubule-associated protein 1 A/1B-light chain 3B (LC3B) and sequestosome 1 (P62) are classical autophagy-related proteins that we then assessed the level of autophagy. After 48 h of stimulation with high glucose, LC3B levels in HUVECs were significantly reduced, while a significant increase in P62 levels was observed (Fig. 5B). We also assessed angiogenesis levels 48 h after stimulation of HUVECs. Among them, the tube formation experiment showed that the tube formation ability of HUVECs was significantly weakened under the stimulation of high glucose (Fig. 5C). The wound healing assay showed that high glucose stimulation significantly reduced the healing rate at 12 h and 24 h (Fig. 5D). Moreover, the level of vascular endothelial growth factor A (VEGFA) protein in HUVECs stimulated by high glucose was significantly reduced (*P =* 0.0189).

### Inhibition of EGLN1 rescues hyperglycemia-induced autophagic suppression and endothelial dysfunction

**Fig. 6:**
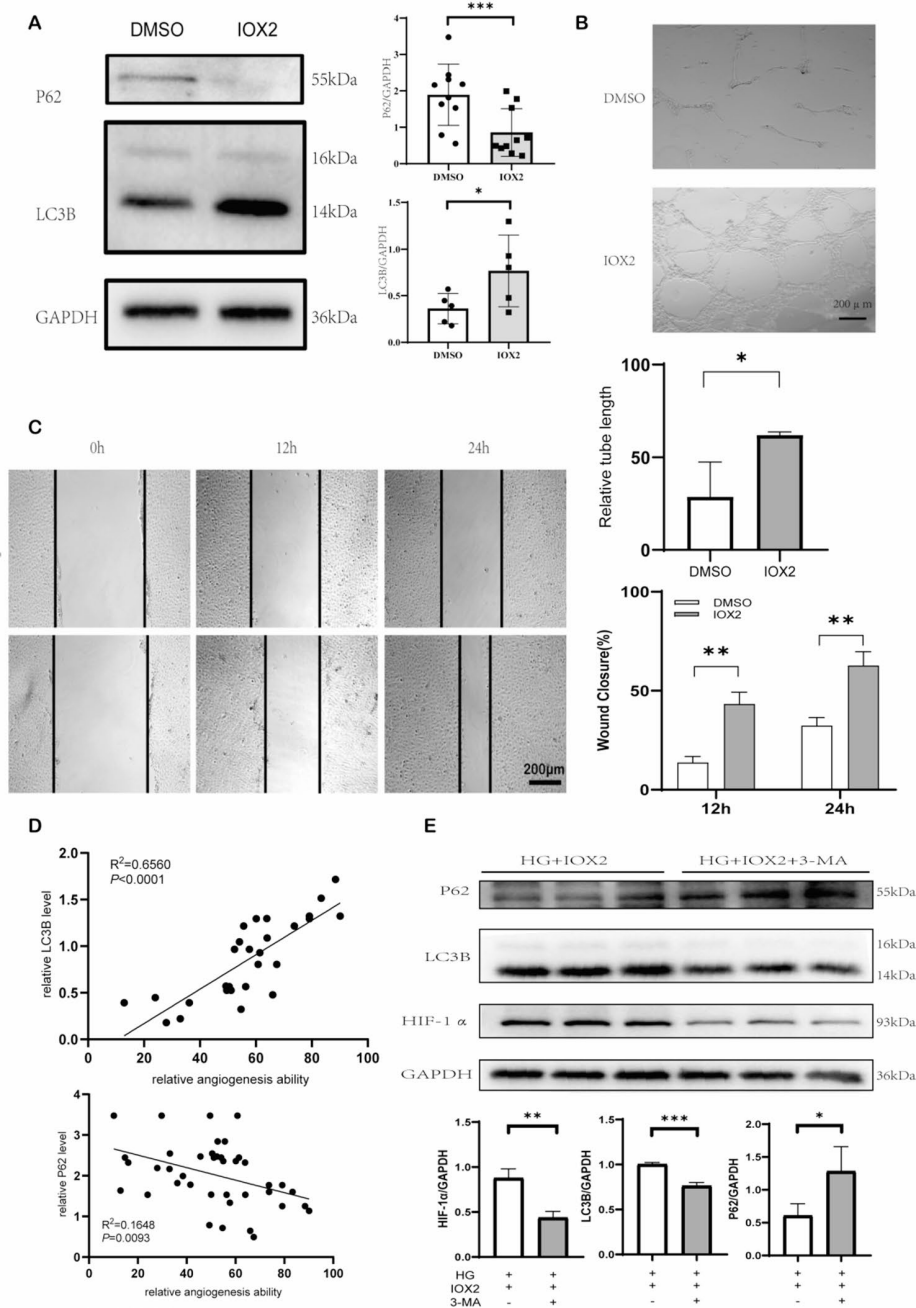
Inhibition of EGLN1 rescues hyperglycemia-induced autophagic suppression and endothelial dysfunction. (**A**) Representative western blot images and relative analysis of LC3B-II/LC3B-I ratio (*n* = 5) and P62 (*n* = 10) protein levels in HUVECs, HUVECs were exposed to 30 mM glucose and simultaneously added in equal volumes of DMSO or IOX2 (EGLN1 inhibitor, at a concentration of 50 µmol/L) for 48 h. (**B**) Representative images and quantification of tube formation assay in HUVECs stimulated by high glucose combined with DMSO or IOX2, scale bar = 200 μm (*n* = 3). (**C**) Representative images and quantification of wound healing assay in HUVECs stimulated by high glucose combined with DMSO or IOX2 at 12 and 24 h, scale bar = 200 μm (*n* = 3). (**D**) Scatter plot of correlation analysis between LC3B or P62 expression and angiogenesis capacity (including tube formation and wound-healing assessment). (**E**) .WB images and relative statistical analysis of autophgy-related proteins (LC3B and P62) and EGLN1 downstream protein (HIF-1α) in HUVECs stimulated by high glucose after IOX2 intervention with or without 3-MA. (*n* = 3). All data represent the mean ± SEM. *P < 0.05, **P < 0.01, ***P < 0.001.

In order to evaluate the effect of EGLN1 inhibitor, inhibitor of hypoxia-inducible factor prolyl hydroxylase 2 (IOX2) on HUVECs stimulated by high glucose, IOX2 or dimethyl sulphoxide (DMSO) were added under the stimulation of high glucose. We found that IOX2 (50 µmol/L) produced a rescue effect on autophagy compared to DMSO alone, in which IOX2 increases LC3B levels while decreasing P62 expression at high glucose (Fig. 6A). And EGLN1 knockdown was performed in HUVECs using siRNA transfection. It’s functional effects on HIF-1α and autophagy markers (LC3B, P62) were confirmed by Western blot (see Supplementary Material Fig.S1). Considering that EGLN1 inhibitors have been put into clinical use, there may be more prospects for clinical translation, so we then proceeded with IOX2 for our research.

Next, we were also stimulated with high glucose for 48 h, and at the same time, IOX2 (50 µmol/L) or DMSO was added as a control. It was found that the tube formation (Fig. 6B) and wound healing capacity (Fig. 6C) of HUVECs was significantly improved. To further illustrate the relationship between autophagy levels and angiogenesis, we plotted the expression levels of LC2B and P62 against angiogenesis capacity (including relative tube length and scratch healing rate). We found that there was a positive correlation between the expression level of LC3B and angiogenesis capacity, while there was a negative correlation between P62 (R^2^ = 0.656, 0.1648 respectively) (Fig. 6D). After the addition of autophagy inhibitor 3-methyladenine (3-MA) with IOX2, LC3B was significantly reduced and P62 accumulation was significant and statistically significant (*P =* 0.0007, *P =* 0.0492). However, HIF-1α, a downstream molecule of EGLN1, showed a significant decreasing trend after the addition of autophagy inhibitors (*P =* 0.0036) (Fig. 6E).

## Discussion

We hope to identify key target molecules related to angiogenesis in diabetic cardiomyopathy, and then provide ideas for clinical intervention. Drugs such as apelin^13,28,29^, adipsin^30^, and cilostazol^31^ have been found to improve angiogenesis in DCM. Recent studies have confirmed that Shexiang Tongxin Dripping Pill has a definite therapeutic effect on diabetic coronary microcirculatory dysfunction, and mechanistic studies have found that it is related to the promotion of angiogenesis through the VEGF/endothelial nitric oxide synthase (eNOS) signaling pathway^32^. While these studies have proposed many potential therapeutic targets, there is still a lack of specific interventions targeting angiogenesis in diabetic cardiomyopathy.

In related studies of diabetic cardiomyopathy, we have noted that autophagy is involved in the occurrence and progression of DCM and is a potential pharmacological target for DCM^22^. A growing number of studies have found that the level of autophagy is reduced in high-glucose-stimulated cell models and animal models of diabetes: high glucose induces decreased activity and autophagy activation in cardiomyocytes^33^ and cardiac fibroblasts^34^. Regulating the level of autophagy can play a therapeutic role, and metformin and resveratrol have been found to alleviate apoptosis and play a protective role by enhancing autophagy^35–37^. Recent studies have demonstrated that the neuregulin-4 and CREG1-FBXO27-LAMP2 pathways promote cardiomyocyte autophagy and thus alleviate DCM^38,39^.

Finally, we combined multiple data sets and found that *EGLN1* may be a risk gene for cardiovascular and metabolic diseases. Recent studies have confirmed that EGLN1 levels are significantly positively correlated with visceral adipose tissue, body mass index, glycated hemoglobin, insulin, triglycerides and metabolic syndrome^40^. Previous studies have found that EGLN1 regulates hypoxia-inducible factor (HIF)’s degradation through hydroxylation modification^15^and EGLN1/HIF can regulate angiogenesis-related genes such as VEGF, Notch ligand and endothelial nitric oxide synthase (eNOS)^41,42^. A recent study found similar results, and EGLN1 was upregulated in diabetic cardiomyopathy after combined bioinformatics and in vitro verification^18^.

Furthermore, we found that IOX2 can enhance the autophagy level of high-glucose-stimulated HUVECs and improve angiogenesis, which may be a therapeutic direction that can be further translated. EGLN inhibitors have been developed for the treatment of renal anemia in patients with chronic kidney disease, including roxadustat, daprodustat, vadadustat, and enarodustat^43–45^. Beneficial modulatory effects of these drugs on metabolism have been found^46,47^, and roxadustat has been shown to improve left ventricular hypertrophy in hemodialysis patients compared with erythropoietin (EPO)^48^. Recently, roxadustat has been found to enhance the PI3K/AKT/Nrf2 pathway and regulate oxidative stress in cardiomyocytes induced by high glucose, thereby showing cardioprotective effects in diabetic mice^49^. This shows the potential of EGLN1 inhibitors in the treatment of angiogenesis damage in diabetic cardiomyopathy, and our study also verified the important role of EGLN1 in angiogenesis in DCM through the database screening of target molecules, and confirmed that IOX2 can act on the autophagy process to exert angiogenesis protection.

But inevitably, this study also has some limitations. Our research on autophagy levels and EGLN1 inhibitors has focused on the cellular level, which may overlook a lot of overall information, especially the body’s large and delicate regulatory system that can act on physiological and pathological processes. Secondly, due to the limited laboratory conditions and resources currently available, our validation method is relatively simple, and the sample size needs to be further expanded, and more studies are needed to confirm the role of EGLN1 in DCM angiogenesis injury. Finally, we used STZ combined with HFD for DCM mouse modeling, but as our previous analysis showed, EGLN1 also plays an important role in metabolic diseases, so we cannot completely rule out the effects of STZ and HFD on EGLN1 levels, and more models are needed to verify the accurate role in the later stage.

## Conclusion

In summary, this work provides the first preclinical evidence linking EGLN1 dysregulation to microvascular pathology in diabetic hearts, offering a rationale for developing targeted interventions to mitigate cardiovascular morbidity in diabetes. Further investigations into tissue-specific EGLN1 signaling and translational validation of autophagy-based therapies are warranted to advance clinical applications.

## Methods

### Data preparation

In the GEO database, high-throughput sequencing data related to “diabetic cardiomyopathy”, “diabetic vascular injury”, “diabetic angiogenesis” and other related data were searched. The sequencing data of basic experimental samples and clinical samples of diabetic vascular injury were identified, respectively. Among them the GSE241565 dataset^50^ includes sequencing data of HUVECs stimulated by high glucose; The GSE215979 dataset^51^ contained sequencing data of heart tissue extracted from normal control and diabetic model mice. Besides, sequencing data of diabetic patients with microvascular complications and their matching controls were extracted from the GSE43950 dataset. In addition, Angiogenesis-related genes in humans were retrieved from the GeneCards database (https://www.genecards.org/), whereas murine counterparts were acquired from the Mouse Genome Informatics (MGI) database (https://www.informatics.jax.org/).

### Differential expressed genes screening and functional enrichment analysis

GSE43950 datasets were analyzed using online GEO2R, in which the threshold value of GEO2R analysis was corrected *P <* 0.05, and the adjusted P value of the analysis was calculated by Benjamini & Hochberg (False Discovery Rate), based on R 4.2.2 and limma 3.54.0. Then top 100 differentially expressed genes were analyzed and clustered using the STRING database (https://cn.string-db.org/). Next, DAVID Bioinformatics (https://davidbioinformatics.nih.gov/) was used to perform functional and pathway enrichment analysis. KEGG pathway maps are copyright protected by Kanehisa Laboratories^52,53^.

### Analysis of key differential genes

Three sequencing datasets (included cellular, animal and human sequencing data) were combined to screen the key differentially expressed genes associated with angiogenesis in diabetic cardiomyopathy. Finally, the expression of key genes in cardiovascular and metabolic diseases and related clinical disease risk analysis were extracted through human protein atlas database (HPA) (https://www.proteinatlas.org/) and proteome-phenome atlas (PPA) (https://proteome-phenome-atlas.com/).

### Animal model

All C57BL/6 N mice (male, 7 weeks, 21–25 g) were purchased from Vital River and were randomly divided into diabetes mellitus (DM) group and negative control (NC) group after adaptive feeding. All mice were housed in a quiet environment, with 12 h of alternating light and dark and regular disinfection and sterilization. Mice had free access to food and clean drinking water. Diabetic model mice were fed a high-fat diet (HFD) and injected intraperitoneally with STZ (30 mg/kg, MedChemExpress, USA) was dissolved in sodium citrate buffer (pH4.5, sterile, Solaibio, C1013) for 4 consecutive days^54^. The percentage of calories (kcal%) was 19.8% protein, 35.2% carbohydrate and 45.0% fat. All experimental protocols involving live animals were approved by the Reseach Ethics Committee of the second hospital of Hebei Medical University (2024-AE071). The handling of mice and experimental procedures were conducted in accordance with the relevant guidelines and regulations established by the committee. Mice were anesthetized and euthanized by isoflurane.

### Immunofluorescence staining

Immunofluorescence staining (CD31) of mouse heart tissue was performed to assess angiogenesis. Sections of OCT-embedded fresh heart tissue (5 μm thick) were plated on glass slides. Staining was first equilibrated at room temperature for 10 min and then washed 3 times in PBS. This was followed by 30 min of blocking with 5% goat serum at room temperature, CD31 primary antibody (1:50, mouse primary antibody from GeneTex. GTX20218) overnight at 4 °C. The next day. Fluorescent secondary antibodies (1:100) were incubated for 1 h at room temperature in the dark, and finally, images were observed and captured under a confocal microscope (Olympus).

### Cell culture

HUVECs used in this study were kindly provided by the Department of Biochemistry and Molecular Biology of Hebei Medical University and cultured in DMEM containing 25 mM glucose, 10% FBS, and 1% penicillin/streptomycin. For high-glucose conditions, 30 mM glucose was used. After 48 h of culture under normal or high-glucose conditions, cells were harvested. Incubator conditions were maintained at 37 °C with 5% CO_2_ and 95% humidity.

In order to evaluate the effect of EGLN1 inhibitor (IOX2, HY-15468, Medchemexpress, USA) on HUVECs stimulated by high glucose, HUVECs were stimulated with high glucose, and then IOX2 (dissolved in DMSO, 50 µmol/L) or the equivalent volume of DMSO was used to continue the experiment. Subsequently, 3-MA (50 µmol/L, HY-19312, Medchemexpress, USA), was used to further verify whether IOX2 exerts a downstream effect by regulating the level of autophagy.

### Wound healing and tube formation assay

To evaluate angiogenic potential, HUVECs were seeded in 6-well plates and stimulated with low- or high-glucose for 48 h. A scratch was made using a 200-µL pipette tip, and cells were rinsed with PBS. Culture medium was replaced with serum-free DMEM, and images were captured at 0 h, 12 h, and 24 h. Scratch migration length was measured using ImageJ software, with at least three measurements per well to calculate the migration rate. Matrigel (BD Biocoat 356234, Corning, USA) was chilled overnight and applied to 24-well plates. HUVECs were then seeded and cultured in serum-free DMEM. Tube formation was observed and photographed using an inverted microscope. Quantitative analysis was performed using the ImageJ “Angiogenesis Analyzer” plugin.

### WB

HUVECs were lysed with RIPA buffer containing 1% PMSF, followed by centrifugation to collect the supernatant. Protein concentration was determined using a BCA kit (Solaibio, China). Denatured proteins were resolved by 10% SDS-PAGE and transferred to a PVDF membrane (Millipore, Burlington, MA, USA). The membrane was blocked with 5% milk at room temperature for one hour, incubated with primary antibodies against EGLN1 (1:1000, Upingbio, YP-mAb-17870, China), P62 (1:500, Wanleibio, WL02385, China), LC3B (1:1000, Cell Signaling Technology, Cat# 2775, USA), HIF-1α (1:500, Wanleibio, WL01607, China), VEGFA (1:500, Wanleibio, WL00009b, China) and GAPDH (1:2000, Huabio, R1210-1, China), and then with an HRP-conjugated secondary antibody. Protein bands were visualized using a Bio-Rad gel imager and analyzed with ImageJ software. And all original images of the western blot were provided with supplemental material (see Supplementary Material Figs. 4–6S).

### ELISA

Samples and reagents were equilibrated to room temperature for 60 min. Pre-diluted wash buffer was prepared per manufacturer instructions. Pre-coated strips were loaded with 50 µL of standards (125–4000 pg/mL) or test samples, followed by addition of 100 µL/well HRP-conjugated detection antibody. After 60 min incubation at 37 °C in darkness, plates underwent five automated wash cycles (300 µL/cycle) with thorough blotting. Freshly mixed substrate solution (100 µL/well) was added and incubated for 15 min at 37 °C. Reactions were terminated with 50 µL stop solution, and absorbance was measured at 450 nm. Sample concentrations were calculated using four-parameter logistic regression (validated by R^2^ > 0.99).

### qPCR

Mice cardiac tissues were homogenized in liquid nitrogen. Total RNA was extracted using TRIzol reagent, and cDNA was synthesized from 1 µg RNA per manufacturer’s protocol (A505, Exongen biotechnology, China). qPCR was performed with SYBR Green Master Mix (A411, Exongen biotechnology, China). Reactions included: 95 °C for 5 min; 40 cycles of 95 °C/10 s, 60 °C/30 s; followed by melt-curve analysis. Gapdh served as the endogenous control. Relative expression was calculated via the 2^−ΔΔCt^ method. All samples were run in triplicate. Primers are constructed by the Generay biotechnology company (Shanghai, China), and the details are as follows: Egln1-F: CCTGGATCGAGGGCAAAGAG, Egln1-R: CGGACATAGCCTGTTCCGTT; Gapdh-F: CATCACTGCCACCCAGAAGACTG, Gapdh-R: ATGCCAGTGAGCTTCCCGTTCAG.

### Statistical analysis

All data were presented as mean ± standard error of the mean (SEM), and the statistical analysis was conducted by GraphPad Prism 8.0 (GraphPad Software, Inc., San Diego, CA, USA). Statistical differences between the two groups were analyzed using t-test. In addition, correlation analysis was performed using Pearson and *P* < 0.05 was considered statistically significant.

## Supplementary Information

Below is the link to the electronic supplementary material.

Supplementary Material 1

## Data Availability

The datasets used and/or analyzed during the current study available from the corresponding author on reasonable request.

## Declarations

### Accordance

We confirmed that all experiments in this study were performed in accordance with the relevant guidelines and regulations.

### Arrive

All the procedure of the study is followed by the ARRIVE guidelines.
