# Supplementary material for: EGLN1 inhibition reverses angiogenesis impairment in hyperglycemia by activating autophagy
Source: Sci Rep. 2025 Oct 14;15:35804. doi: 10.1038/s41598-025-19745-6 (PMC12521508; doi:10.1038/s41598-025-19745-6)
Supplement: Supplementary file 1 — Supplementary Material 1 [file 41598_2025_19745_MOESM1_ESM.docx]

Supplementary Material

Manuscript ID: bbf86a55-a234-4f32-af70-143c3db75044

Title: Targeting EGLN1 rescues hyperglycemia-impaired angiogenesis via autophagy-mediated endothelial repair

Authors: Fengli Hu^1,2^, Zheng Li^2^, Ying Li^2^, Yaxin Zhi^1,2^, Ting Tang^1,2^, Pengfei Wang^1,2^, Ling Xue^1,2,*^

**Section 1: Full-length Western Blots**

**
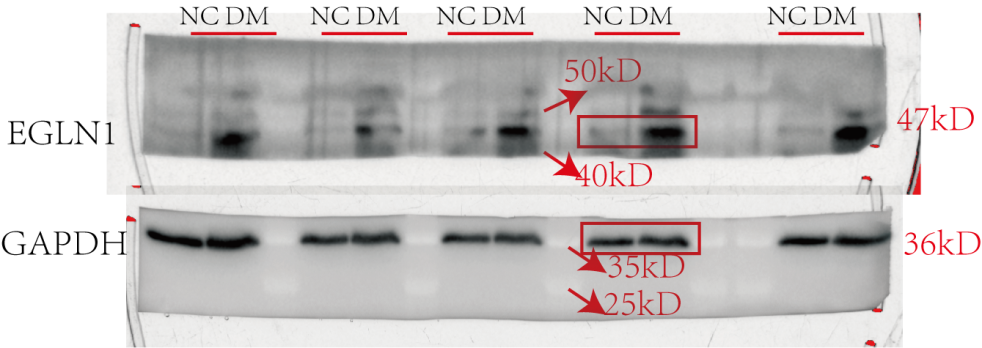
**

**Fig.4S** Representative western blot images and relative analysis of EGLN1 on hearts of NC and DM mice.


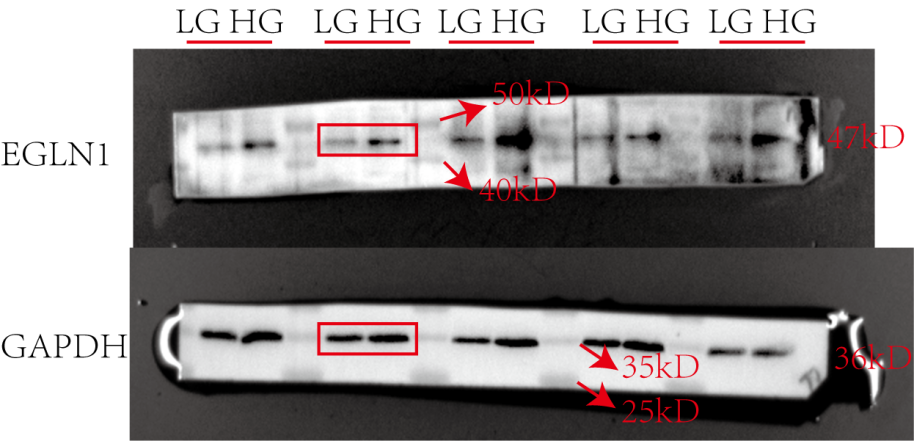


**Fig.5S**(A) Representative western blot images and relative analysis of EGLN1 protein levels in HUVECs exposed to 30 mM glucose for 48 h compared with normal glucose controls (5.5 mM).


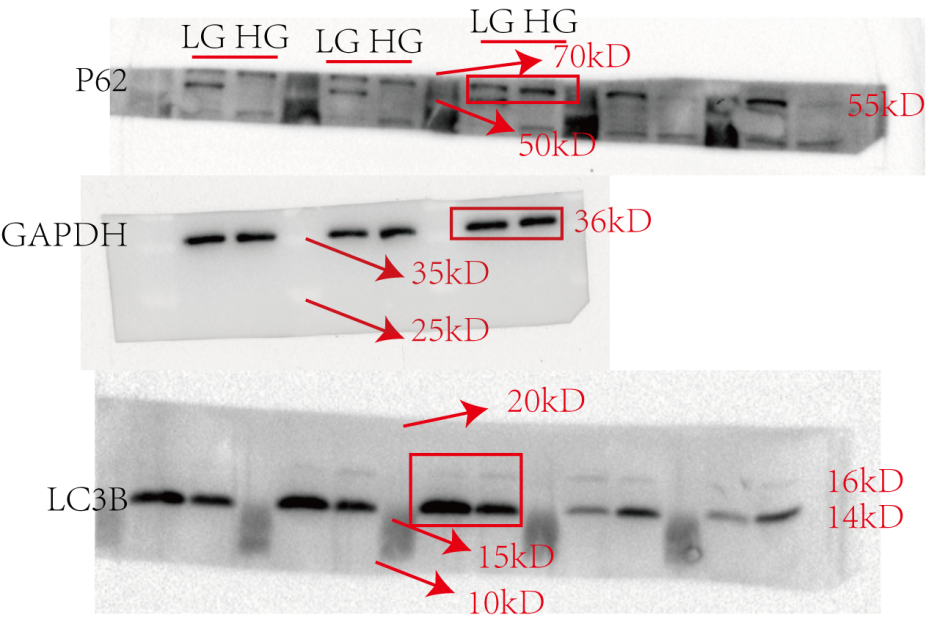


(B) Representative western blot images and relative analysis of LC3B-II/LC3B-I ratio (n=5) and P62 (n=6) in high glucose-treated HUVECs.


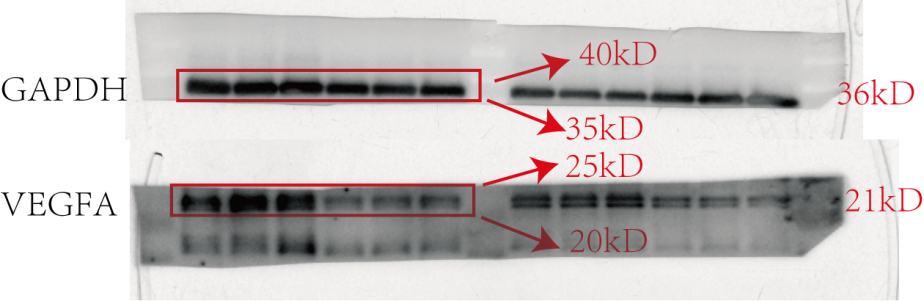


(E) Western blot images and relative analysis of VEGFA in high glucose-treated HUVECs.


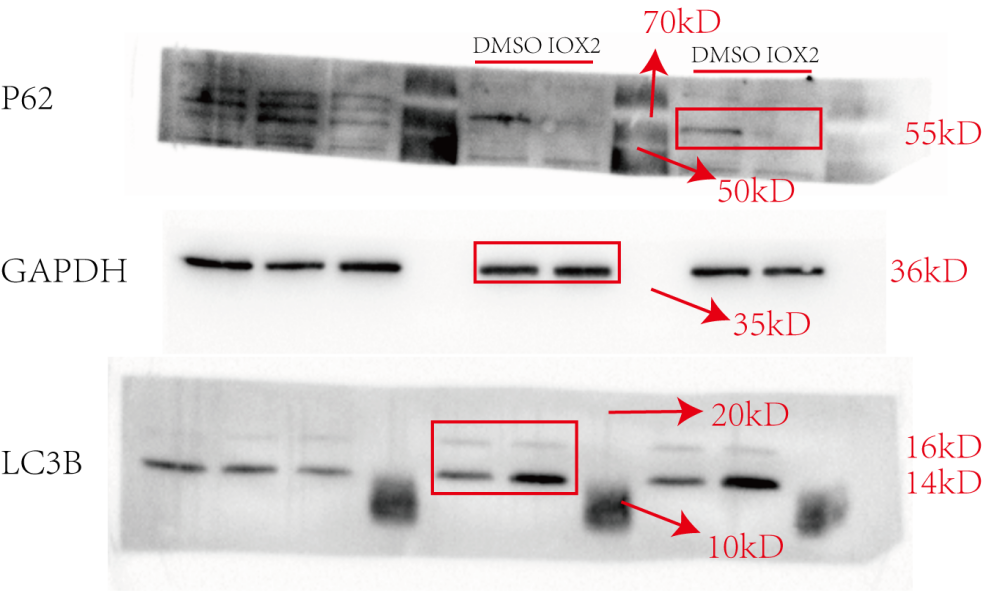


**Fig.6S**(A) Representative western blot images and relative analysis of LC3B-II/LC3B-I ratio and P62 protein levels in HUVECs, HUVECs were exposed to 30 mM glucose and simultaneously added in equal volumes of DMSO or IOX2 (EGLN1 inhibitor, at a concentration of 50 μmol/L) for 48 h.


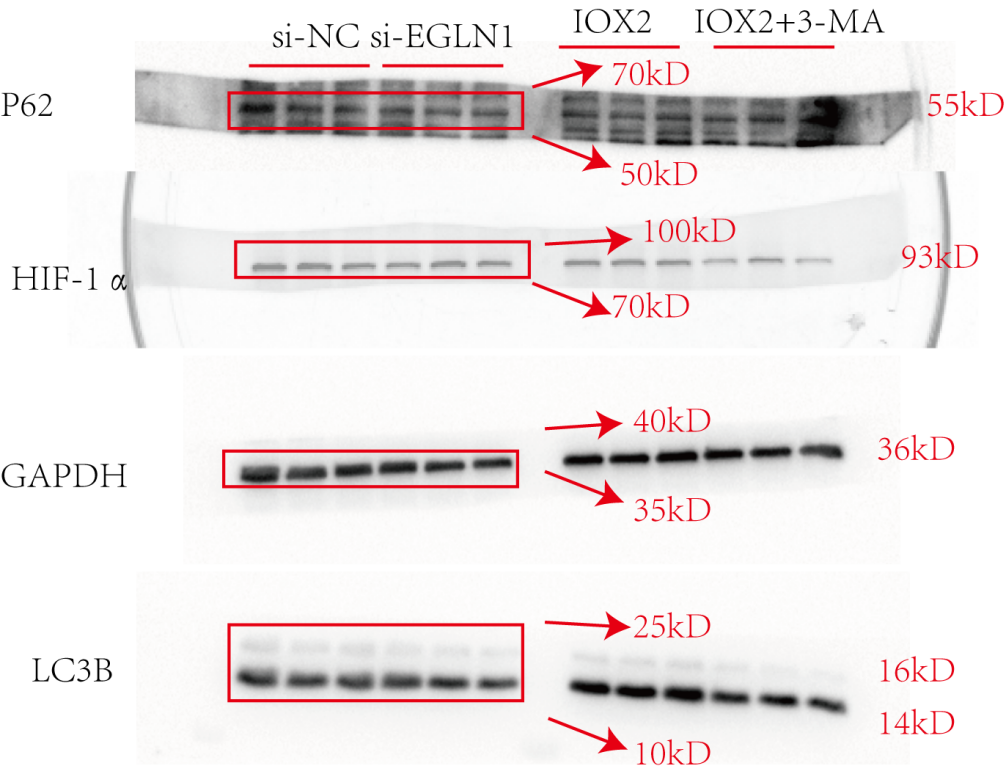


(E) .WB images and relative statistical analysis of autophgy-related proteins (LC3B and P62) and EGLN1 downstream protein (HIF-1α) in HUVECs stimulated by high glucose after IOX2 intervention with or without 3-MA.

**Section 2: Extended Methods**

EGLN1 gene intervention (siRNA transfection of HUVECs)

(1) Plating was performed the day before transfection, and the cells were ready for transfection when they reached about 70% confluency; Cells were washed 3 times in PBS buffer prior to transfection, followed by 4 mL of serum-free, bispecific antibody-free basal medium in a 6 cm dish. (2) The siRNA (200 pmol, Genepharma, China) and transfection reagent (10 μL, Lipofectamine 3000, L3000015, thermofisher, USA) were diluted in serum-free and bispecific antibody-free basal medium, and the transfection reagent was incubated at room temperature for 5 min, and then siRNA was added and incubated at room temperature for 15 min. (3) Add the incubated reagents drop by drop and evenly into the cell culture wells containing fresh serum-free medium, and gently shake the culture plate several times before and after addition, so that the complexes are evenly distributed in the wells. (4) Return the plate to a 37°C, 5% CO2 cell culture incubator and incubate for 4-6 h. (5) After the end of incubation, carefully aspirate the transfection medium containing the complex, immediately add the complete medium to continue the culture, and collect the cells for Western blot to detect the protein level after 48 h.

The sequence information used is as follows:

Negative Control

sense: UUCUCCGAACGUGUCACGUTT

antisense: ACGUGACACGUUCGGAGAATT

Human EGLN1 siRNA (EGLN1-Homo-1629)

sense: GAGGGUUGAACUCAAUAAATT

antisense：UUUAUUGAGUUCAACCCUCTT

It was found that EGLN1 knockdown had an effect on both HIF-1α and autophagy-related protein P62 (Fig.S1): HIF-1α expression was significantly increased after EGLN1 knockdown (*P* = 0.0477). However, there was no change in LC3B and a decrease in P62, suggesting that the autophagic flux was enhanced after knockdown of EGLN1, but no statistical significance was found (*P* = 0.8918, *P* = 0.0562).

**
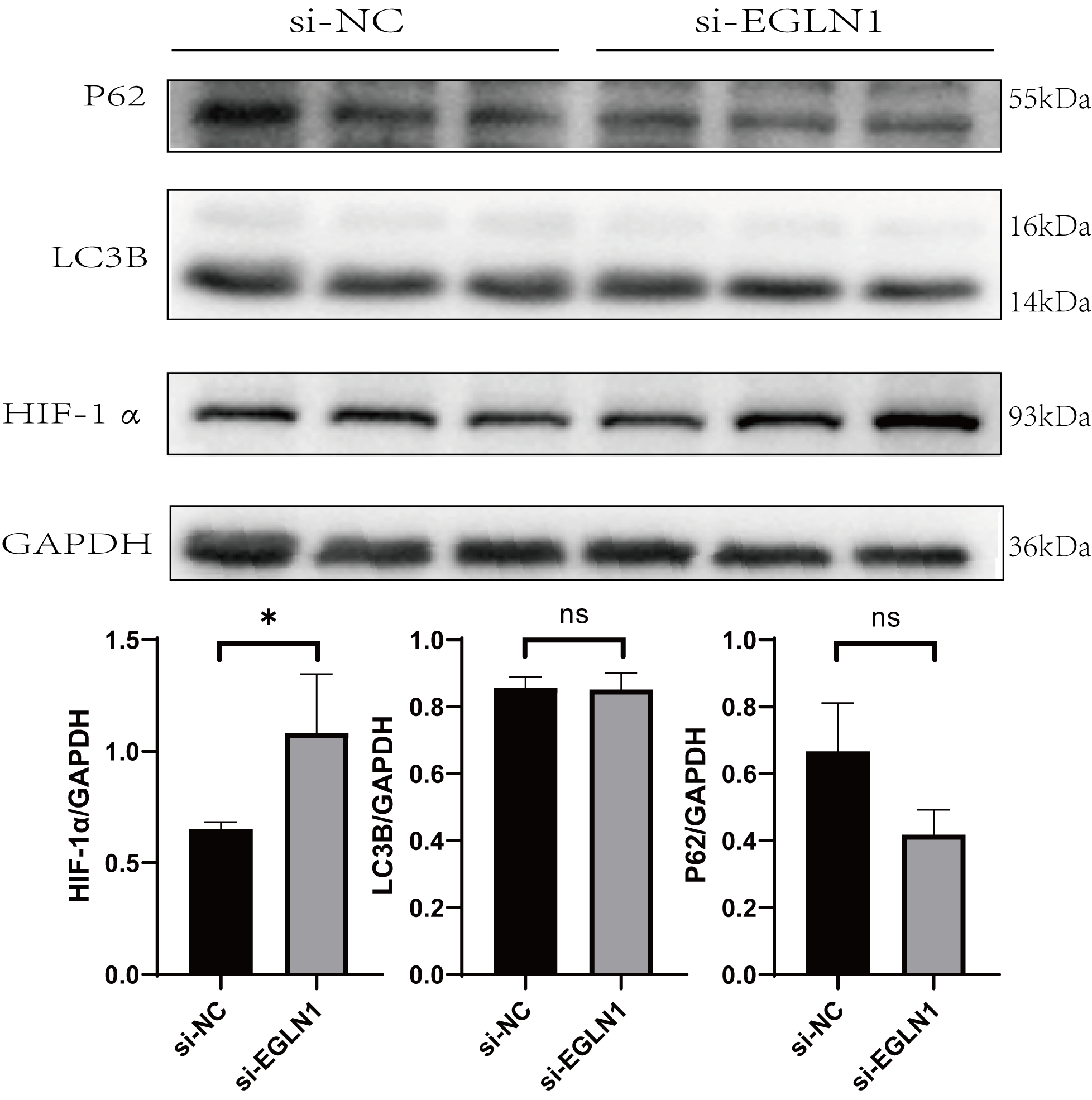
**

Fig.S1. Effects of EGLN1 knockdown on autophagy and downstream protein levels in HUVECs under high glucose stimulation and correlation analysis.

WB images and relative statistical analysis of autophgy-related proteins（LC3B and P62） and EGLN1 downstream protein（HIF-1α） in HUVECs stimulated by high glucose after transfection of EGLN1 small interfering RNA or negative control.(n = 3) Data represent the mean±SEM. **P* < 0.05. NC, negative control; EGLN1, egl-9 family hypoxia inducible factor 1; LC3B, microtubule-associated protein 1A/1B-light chain 3B; P62, sequestosome 1; HIF-1α, hypoxia-inducible factor 1-α; HUVECs, human umbilical vein endothelial cells.
